# A Survey of Management Practices That Influence Performance and Welfare of Dairy Calves Reared in Southern Brazil

**DOI:** 10.1371/journal.pone.0114995

**Published:** 2014-12-15

**Authors:** Maria J. Hötzel, Cibele Longo, Lucas F. Balcão, Clarissa S. Cardoso, João H. C. Costa

**Affiliations:** Laboratório de Etologia Aplicada e Bem-Estar Animal, Departamento de Zootecnia e Desenvolvimento Rural, Universidade Federal de Santa Catarina, Florianópolis, Brazil; ETH Zurich, Switzerland

## Abstract

Here we report dairy calf management practices used by 242 smallholder family farmers in the South of Brazil. Data were collected via a semi-structured questionnaire with farmers, inspection of the production environment and an in-depth interview with a sample of 26 farmers. Herds had an average of 22.3 lactating cows and an average milk production of 12.7 L/cow/day. Calves were dehorned in 98% of the farms, with a hot iron in 95%. Male calves were castrated in 71% of the farms; methods were surgery (68%), emasculator (29%), or rubber rings (3%). No pain control was used for these interventions. In 51% of the farms all newborn male calves were reared, sold or donated to others; in 35% all newborn males were killed on the farm. Calves were separated from the dam up to 12 h after birth in 78% of the farms, and left to nurse colostrum from the dam without intervention in 55% of the farms. The typical amount of milk fed to calves was 4 L/day until a median age of 75 days. In 40% of the farms milk was provided in a bucket, in 49% with bottles, and in 11% calves suckled from a cow. Solid feeding in the milk-feeding period started at a median age of 10 days. Calves were housed individually in 70% of the farms; in 81% of the farms calves were housed in indoor pens, in 6% in outdoor hutches and in 13% they were kept on pasture. Diarrhoea was reported as the main cause of calf mortality in 71% of the farms. Farmers kept no records of calf disease, mortality, or use of medicines. Changing the scenario identified in this survey is essential to support the sustainable development of dairy production, an activity of great economic and social relevance for the region.

## Introduction

In the state of Santa Catarina, the fifth largest and fastest growing Brazilian dairy producer [1], 87% of the milk is produced in family farms of up to 50 ha [2]. Milk generates income to an estimated 50 thousand families and employment opportunities to many others [3], [4]. For these reasons the milk production chain is considered among the most important activities for family farming by the Brazilian Federal Government, which supports it through a variety of programs [5].

Animal welfare is recognised as an essential component of the social pillar of sustainability for the dairy industry [6], [7]. Societal concerns regarding the ethical treatment of animals have raised the interest in animal welfare in Brazil [8]. The quality of dairy calves' rearing may impact the welfare of the calves, the productive outcome of the future cow and, through heifers' survival indices, the rate of genetic improvement of the herd. In recent years there has been important scientific advancement on our understanding of several aspects of dairy calf rearing that influence their growth, health and wellbeing. Neonatal calf care, feeding and housing practices, the fate of newborn male calves and pain inflicting procedures practiced routinely in dairy farms are considered some of the most critical areas in calf rearing that impact animal welfare [6], [9], [10].

Colostrum management is key to calf health, survival and welfare, as inadequate intake leads to increased mortality and risk of diseases such as diarrhoea and respiratory illnesses [11]. Feeding calves known amounts of colostrum soon after birth is more effective [12]–[14] than leaving them to suckle for colostrum from the dam without supervision, which increases the risk of failure of passive transfer (defined as a circulating immunoglobulin concentration bellow 10.0 g/L [15]). Besides conferring immunity to the neonate, colostrum stimulates the maturation and function of the gastrointestinal tract in neonate calves, enhancing the absorptive capacity of the gastrointestinal tract [16].

Neonate management often includes early separation from the dam. A recent study involving North American citizens [17] suggests that most people not familiar with the dairy industry oppose this practice, which contrasts with a more favourable view among farmers, veterinarians and other dairy professionals. The views of the latter are underpinned by perceptions that early separation prevents disease transmission and reduces separation stress in the cow and calf [10]. Also based on these reasons, early separation is widely recommended in the Brazilian technical literature [18], [19]. However, contact with the dam during early life may have several advantages, like favouring calf growth and health and reducing abnormal behaviours [16], [20]–[23].

Until recently there has been some controversy in the literature regarding the long term effects of early growth on the onset of puberty, first lactation and milk production – with some studies reporting no relationship [24], [25] and others reporting a positive association [26], [27]. A recent meta-analysis of 12 published studies concluded that increasing nutrient intake from milk or milk replacer in preweaned calves has overall positive impacts on first lactation milk production [28]. However, dairy calves are often provided limited milk allowances, which besides potentially restricting growth, reduce health [29], increase risk of perinatal mortality [30], and are associated with behaviours indicative of hunger [31]–[33].

Most preweaning calves are housed individually in farms in the United States [34], Canada [35], and Europe [36]. Group housing may improve postweaning growth [37], [38] and the development of cognitive and social skills [39]–[41]. The preference for individual housing stems from the idea that this management can reduce the incidence of cross-suckling and disease transmission [10]. These problems may be reduced or overcome with some management practices. For example, health problems in group-housed calves seem to be mostly associated with large groups (see review by Von Keyserlingk and Weary [10]), which in turn may be related to poorer hygiene than in single housing systems. Cross-suckling may be prevented by using teats, instead of buckets to supply milk to calves, added to adequate milk allowances that prevent hunger [9], [32].

Scientific information gathered in on-farm surveys can be valuable to guide public policy, and research and extension programs aiming to support on-farm adoption of best practices to improve animal welfare and productivity. Surveys of dairy calf rearing practices conducted in North America [34], [35], [42] and European farms [30], [36], [43], [44] have identified the main practices that need to be improved in order to achieve productive and animal welfare goals. Similar information on dairy calf rearing practices used by Brazilian family farmers is not available. In a recently published paper [45] we presented data from an on-farm survey carried out in the South of Brazil pertaining to lactating cattle welfare and productivity. In this paper we present data covering aspects of calf rearing from birth to weaning in farms of the same region. We evaluated aspects of the living environment of the animals and management practices used, and interviewed a subsample of the farmers, aiming to understand the reasons behind their choices of practices.

## Materials and Methods

### (a) Ethics Statement

This study was approved by the Ethics Committees on Research on Humans and Animals of the Universidade Federal de Santa Catarina (Protocol numbers, respectively: 11837612.1.0000.0121 and PP00859). Prior to the initiation of the interviews, each participant provided informed consent and was made aware that his/her identity would remain anonymous.

### (b) General Methodology

We carried out a survey of dairy calf management practices used in dairy smallholdings in Santa Catarina state, Brazil. Farms (n = 242) were distributed in 29 different municipalities in the western (n = 124) and in the southern (n = 118) regions of the state. These are the first and third main dairy producing regions and account for 79.4% of the milk produced in the state [1].

Santa Catarina (located between 25°57′41″S and 29°23′55″S) has a humid subtropical climate (Köppen climate classification Cfa and Cfb). It is characterized by humidity over 75% all year round, with hot, humid summers and generally mild winters. The region presents a historical monthly mean temperatures ranging from 23°C in January – summer in the southern hemisphere – to 14°C in July. Mean annual cumulated rainfall is 1,900 mm.

Farms were visited in the Spring/Summers 2010–2011 to 2012–2013. Farm recruitment was done as described in Costa et al [45]: advisors (extensionists, veterinarians or agricultural technicians working for private or public extension bodies) were asked to indicate three to five farms that represented the main farm types found in the region in terms of farm and herd size and production system, as described by Lorenzon et al [46]. Some of the farms were recruited using a snowball technique, whereby farmers that had agreed to participate were asked to recommend other farmers that would be willing to participate. Farmers were contacted by telephone by a member of the research team to make an appointment for a visit on a further occasion.

The on-farm assessment took half of a day and included an hour-long interview with closed and open questions, and an inspection of the environment. Additionally, on a second visit a subset of 26 farmers, from 20 farms, participated in an in-depth interview.

### (c) Inspection of the Environment

The inspection of the calves' rearing environment was done with the aid of a checklist (available in full in [47]) to assess the type of management (individual or in groups), the type of housing (pens, pasture or hutches), and hygiene (clean: no presence of faeces and mud patches, regular: presence of some mud and/or some faeces without covering the floor, dirty: presence of mud patches and faeces covering the floor).

### (d) Questionnaire

Face-to-face interviews with farmers were carried out using a questionnaire with multiple-choice and semi-closed questions to collect data on calf rearing husbandry practices and some herd data to characterize the farms [47]. The dairy calf husbandry practices section of the interview first covered data on neonatal care procedures, such as the location of calving, calving protocol, colostrum management and timing of dam-calf. The interview then proceeded with a section on the calf feeding practices, where questions such as the amount and the form of milk delivery, age at weaning and procedure of weaning were collected. The next section covered invasive procedures, including where, at what age and which procedure was used for dehorning, castration, and extra-teat removal. Farmers were asked to give a subjective assessment of the prevalence of disease and mortality (low, regular or high). The last section covered the fate of the newborn male calf, with information pertaining the farmers' decision on keeping or not the male calves, and how they were killed.

### (e) In-depth interviews

A subset of 20 farms participated in in-depth semi-structured interviews. Our aim was to elicit the reasons behind the choice of different calf rearing practices, and not to quantify or compare these reasons across farmers. Standard practice in qualitative research determines that the number of interviews required is reached when no new information is elicited from interviews [48]. In the present study, data saturation was reached at 26 interviews. Ten men and 9 women were interviewed individually, and 5 couples and 2 families were interviewed together.

Farm selection was based on two criteria with equal number of the farms in each group: 1. whether milk production was or was not the main economic activity of the family and 2. the existence of a fully functional system to supply drinking water to cows on pasture, which was used to infer if there was ongoing investment in dairy production [45].

The interviewer asked participants to talk freely about calf rearing practices used in their farms: calf-dam separation, colostrum feeding, milk feeding and weaning, housing, fate of the newborn males. When the issue did not arise spontaneously, the interviewer asked why he/she chose a specific practice.

The interviews were carried out in person by the same trained researcher and all interviews were tape recorded and transcribed in full by this same person. All interviews were carried out and transcribed in Portuguese. The interview transcripts were free coded to identify major themes within and across interviews.

### (f) Data analysis

Descriptive statistics for quantitative variables – average, median, quartiles and range (minimum and maximum) – and frequencies for all categorical variables were individually calculated. All data were analysed with the Hmisc package for the software R (R development, 2013, version 3.0.2). Farms were the experimental unit for all analysis.

## Results

Herd and production data are reported in Table 1. The average herd size and milk production per cow (L/day) of our study population are within the state averages, which are 24 cows [2], and 7.1 to 30 L of milk/cow/day [49], respectively. The herds were Holstein (37%), Jersey (45%) and mixed herd – Holstein×Jersey, Holstein×other or Jersey×other (18%).

**Table 1:** Herd characteristics of dairy farms surveyed in the state of Santa Catarina, Brazil (n = 242).

Variable	Average	Q1	*Median*	*Q3*	*Range*
Total cows (n)	27	14	23	35	5–105
Milking cows (n)	22	11	18	28	5v86
Total milk sold (L/day)	318	100	210	400	30–2,300
Milk production (L/cow/day)	12.7	8.5	11.3	15.8	4–31

### (a) Neonatal care and feeding practices

Neonatal care and feeding practices are reported in Table 2. No specific location for calving was identified in most of the farms; 31% did not have any sort of calving protocol or a monitoring schedule for calving cows. In 55% of the farms calves nursed for colostrum from the dam without intervention.

**Table 2:** Neonatal, feeding and housing practices, as reported by smallholder dairy farmers of Santa Catarina State, Brazil (n = 242).

Variable	Mean (%)
Cow location for parturition	
Close to house	30.5
With herd	69.5
Separation from the dam	
Up to 12 h after birth	71.3
24–72 h	17.8
>72 h*	10.9
Colostrum feeding method	
Calf sucks from dam	54.6
Bottle or bucket fed	45.4
Colostrum feeding duration	
<12 h	41.4
Stores colostrum	7.3
Milk feeding method	
Bottle/nipple	48.7
Bucket	40.4
Foster cow or dam	10.9
Housing type	
Indoors	81.4
Outdoors	18.6
Housing (social)	
Single	70.2
Group	29.8

*Foster cow- and dam-reared calves.

The typical amount of milk or replacer supplied to calves, reported in 72% of the farms, was 4 L per day in two meals, though it varied between 2 and 7 L per day. A variety of feeding methods were reported: 49% of the farmers used bottle feeding, while 8% bottle fed the calves for the first days and thereafter used buckets; 32% used bucket feeding throughout the milk-feeding phase, and 11% used suckling systems: either a nurse cow (8%) or own dam (3%).

A combination of bucket feeding and supply of 4 litres of milk per day or less was found in 39% of the farms; a combination of bucket feeding and individual housing was observed in 29% of the farms visited.

Calves were weaned gradually in 81% of the farms, at varying ages (Table 3). In 91% of the farms preweaning calves were offered concentrate. Also, calves were offered silage in 48%, hay in 43%, and both silage and hay in 15% of the farms; altogether, calves received some roughage in 71% of the farms. Calves' ages when each type of solid feed started being offered are reported in Table 3. Drinking water was freely available to calves in 79% of the farms; on the rest of the farms, calves received water two or three times a day in buckets.

**Table 3:** Timing of calf management practices, as reported by smallholder dairy farmers of Santa Catarina State, Brazil (n = 242).

Variable	Average	Q1	*Median*	*Q3*	*Range*
Calf-cow separation (h after birth)	12.6	0	12	12	0–72
Weaning (days after birth)	81.7	60	75	90	25–270
Age at dehorning (days after birth)	76.2	60	60	90	1–547
Age at castration (days after birth)	204.3	90	180	360	1–720
Concentrate (days after birth)*	14.5	7	10	15	1–90
Silage (days after birth)*	86.0	30	60	120	1–365
Hay (days after birth)*	22.5	7	15	30	1–160

*Age of caves when feed started to be offered.

### (b) Housing

Most calves were housed individually (Table 2). Some calves were reared indoors, in individual (56%) or group pens (26%); others were reared on pasture tied by the neck (9%), in individual hutches (6%), or in collective paddocks (3%). Except for two farms that had the calves nursing on pasture (with nurse cows or own mother), in farms that used suckling systems the calves were housed in pens inside sheds, either in groups (70%) or individually (30%).

Calf housing was scored as “dirty” in 6% of the farms, “regular” in 78%, and “clean” in 6%.

### (c) Painful procedures: dehorning, castration and extra teat removal

Three common elective, painful surgical procedures were carried out in the surveyed farms: dehorning (98% of the farms), castration (79% of the farms), and extra-teat removal (31% of the farms), with procedures carried out at varying ages (Table 3).

The most common dehorning method was hot cautery, used in 95% of the farms, followed by caustic paste (4.5%), and scoop amputation (0.5%). Age at dehorning varied: in 17% of the farms calves were dehorned during the first month of age; in 42% of the farms dehorning between 1 and 2 months, and in 40% between 2 and 6 months. Only 14% hired a veterinarian to perform the procedure; a family member in 76%, a neighbour, a relative or a friend dehorned the calves in 5% of the farms and an employee in 5%.

Surgical castration was the method used in 68% of the farms, followed by the emasculator, used in 29% and rubber rings, used in 3% of the farms. A veterinarian performed the procedure in 16% of the farms (59% of these with the emasculator, 41% surgically), a member of the family, an employee or an acquaintance in 81% (24% of these with the emasculator, 73% surgically and 3% with rubber rings). An advisor, usually an agricultural technician, performed surgical castration surgically in 3% of the cases. In 17% of the farms calves were castrated before 60 days, in 46% between 2 and 6 months and in 37% of the farms, after 6 months of age.

Amputation without cauterization was the most common method for extra teat removal, used in 51% of the farms, followed by amputation with cauterization (38%), and rubber ring (10%).

Only one producer reported the use of a sedative for dehorning, and 3.5% of the farmers for castration; in the rest of the farms no pharmacological methods of pain control were reported for the procedures described above.

### (d) Fate of the newborn male calf

In 51% of the farms all the male calves were reared for consumption at the farm, sold or donated to neighbors or acquaintances; 14% killed the newborn male calves that exceeded their capacity to rear or give away to others, and 35% of the farmers reported killing all newborn males on the farm. Farmers reported killing calves with different methods; the most commonly reported was blunt force trauma to the head (80%), and the rest through exsanguination, asphyxiation or unspecified methods.

### (e) Calf mortality and morbidity

Farmers kept no records on calf mortality, diseases, or the use of medicines or visits by a veterinarian. They rated calf mortality as “low” (80%), “regular” (12%) or “high” (8%). Although when asked to list their main herd health concerns only 12 farmers mentioned calf diarrhoea, 71% reported diarrhoea as the main cause of calf mortality. Respiratory diseases were not mentioned by any of the farmers.

### (f) Farmers' reasons behind choice of husbandry practices

The 20 farms where the in-depth interviews were carried out had on average 20 dairy cows per farm, an average milk production of 18L/cow/day, and total daily production of 400L of milk/day. The main practices that were a focus of this survey varied within the group: in 15 farms the calves were separated from the dam up to 12 h after birth, in 4 of them 24 h, and in one at 72 h after birth; in 6 farms calves were left to nurse colostrum from the cow, and in 14 colostrum was fed with buckets or bottles; milk was offered in a bucket in 12 farms, and with bottles in 8 farms; 15 farms housed calves individually and 5 in groups. In 8 farms the newborn male calves were killed shortly after birth; in 12 they were reared for consumption, or sold or given to neighbours who reared them for consumption.

Three major themes were identified within and across interviews regarding the choice of calf management practices: 1) claims of labour, time or economic cost involved in a given practice were presented as a reason for its use at the farm; 2) a practice was considered a tradition by the interviewee and, 3) perceptions regarding benefits or costs of the practice to the animal.

“Reducing labour” or “saving time” were presented by many as reasons to choose or prefer different practices: type of housing (11 farmers), feeding milk from a bucket (10 farmers), choice of age for dehorning (9 farmers), and to separate the calf from the dam soon after birth (7 farmers).

The main reason presented by farmers (8 who culled the calves at birth, and 3 who did not) to justify the need to cull male calves at birth was lack of pasture or the cost of milk. A farmer that did not kill their newborn males but planned to do in the future explained “Not until today, we have never done that, but from now on… if no one wants them, we'll need to kill them. Because everyone has enough (calves), right?…and then, if we hold them, we have few paddocks, little pasture…”.

Tradition was presented as a reason to feed 4 litres of milk (4 farmers), to rear calves individually (3 farmers), to separate the calves at birth from the dam (2 farmers), and to decide to castrate or dehorn calves (one farmer each).

Perceived positive effects on calves' health and growth explained the choice of housing (9 farmers), bottle feeding (6 farmers) and feeding 4 litres of milk per day, which 5 farmers described as an “adequate” amount. Nineteen farmers raised the problem of cross-suckling in collective housing, or presented it as an advantage of individual housing. Fourteen farmers said that avoiding further stress to the calves (4 farmers) or the cows, which caused difficulty to establish the milking (10 farmers), was the reason to separate the calves early from the dam. Reducing the suffering and improving recovery of the calf was cited as a reason to dehorn (8 farmers) and castrate (6 farmers) calves when they were “young”.

## Discussion

Several rearing practices that limit the health, production and wellbeing of preweaning dairy calves were identified in farms in the South of Brazil. Some important concerns identified in this survey were elective surgical procedures performed without pain control, the fate of newborn male calves, inadequate colostrum provision, and low milk allowances provided to calves during the milk-feeding stage. Adding to previous surveys conducted in North America [34], [35], [42] and Europe [30], [36], [43], [44], [50], our data reveals a wide gap between farm practice and best practices (e.g. [9], [51]) or societal expectations [17], [52], [53]. Recognition of the relevance of animal welfare and productive efficiency to the sustainability of the animal industries [7], [54], [55] underscores the interest and importance of identifying factors that influence this outcome and may help change it.

In the following discussion we cover two relevant themes: some contentious practices carried out on farm, and rearing practices that influence preweaning calves' growth, health and survival.

### (a) Contentious calf rearing practices

To our knowledge this is the first study describing the fate of newborn male calves born in dairy farms in Brazil. A large proportion of newborn calves were killed, and this was done with methods recognized as inhumane by the Brazilian Council of Veterinarians [56] and the American Veterinary Medical Association [57]. What is worrisome is that this problem may increase in the near future due to a combination of factors. First, the problem of exceeding males in smallholdings may increase in the region as a result of concentration of production in fewer, larger herds [58]. Additionally, as farmers seek greater milk productivities there is a trend for an increase in the use of genotypes more specialized in milk production [45], which are less desired for meat production. These factors may increase the real or perceived lack of opportunities to make economic use of the growing number of male dairy calves born.

Most calves were dehorned, and young males were castrated with a combination of methods, late age and absence of pharmacological pain management, which altogether are known to cause pain and reduce welfare (dehorning [51]; castration [60], [61]). Data from this survey confirm that the dehorning procedure is carried out following the standard practice introduced by extension in the region in recent decades [62] and currently recommended by extension [19], [63], i.e. with a hot iron and without pain control. The use of adequate pain control is reported in a fraction of dairy farms throughout the world [35], [42], [43], [64], [65], but in our study it was essentially inexistent. Other studies carried out in the region have shown that on-farm adoption of practices to minimize pain and suffering associated with calf dehorning is prevented by negative attitudes of advisors [63] and farmers' lack of awareness of options [66].

Early separation of the newborn calf from the dam was a standard practice in most visited farms. Alternative rearing systems that allow for social contact and suckling from a cow for a longer period, including foster cow and dam rearing, were used by a tenth of the farms. The main justification offered by farmers in this survey to separate calves from the cow early after birth was the extra amount of labour associated with later separation, caused by the stress response of both calf and cow. Changing this practice, which contrasts with public expectations [17], may require that farmers perceive advantages in alternative practices [20], [22], [23], either in terms of benefits to the animals or through consumers' demand.

These contentious practices discussed above may threaten the sustainability of the local dairy industry, an activity of great economic and social relevance in the South of Brazil [1], [4], [67]. The fact that they are a norm, rather than an exception within the industry may not decrease the likelihood of actions from groups interested in protecting animal welfare, as seen in the recent example of a public campaign to ban mulesing sheep, a widespread practice in Australia [68]. Organizations interested in supporting the dairy industry and family farming may work in two directions: first, developing and promoting opportunities for economic uses of male calves, and practical ways to dehorn and castrate calves on-farm with methods that minimize pain and suffering; second, as suggested by Sneddon and Rollin [68], bringing awareness to farmers and the professionals involved in extension of the changing social ethics (e.g., [17], [53]), and market opportunities for ethically produced farm products.

### (b) Practices that influence preweaning calves growth, health and survival: neonatal care, feeding and housing

Newborn calves were left to nurse colostrum from the cow in more than half of the farms; additionally, few farmers supervised calving or stored colostrum. It has been shown that more than a third of the calves do not ingest any colostrum from the dam during the first 6 h after birth [69], [70], which is the optimum period of immunoglobulin absorption [71]. This increases the risk of failure of passive transfer [15]), consequently increasing morbidity and mortality rates [11]. The poor calving and neonate management described in these farms is likely to be associated to high morbidity and mortality rates, but lack of data may prevent farmers to perceive the problem. Creating mechanisms to improve information on preweaning growth, immunity, morbidity, and mortality in calves in the region is needed to bring awareness to these issues.

Most calves in the surveyed farms received up to 4 L of milk or milk replacer per day, or the equivalent to 10 to 15% of body weight. This amount of milk, commonly recommended in the Brazilian technical literature [72], [73], represents approximately half of a calf's *ad libitum* intake [74]. Four litters per day is associated with hunger [32], reduced health and growth [29], even in the relatively smaller Jersey calves [75], [76] found in many farms. The belief that preweaning weight gain has no long term consequences except for calves' survival seems to underlie the recommendations to feed milk amount “to keep alive and in good health, and not to seek high gain weights” [73]. However, the known effects of early growth on first lactation increased milk yield [28] are similar in individuals of high and low genetic merit [27], indicating that the benefits of increasing milk provision to young calves are likely to extend to the cattle genotypes reared in our study region.

Individual housing was found in the majority of farms. When combined with a low milk allowance, a rearing practice common in one fourth of the surveyed farms, single housing reduces play behaviour [39], indicating reduced welfare [77]. Cross-suckling was cited as an important reason for farmers interviewed in our study to prefer single housing. However, although the frequency of cross-suckling is generally greater in group than in single housing [78], it can be controlled with appropriate management [9]. For example, in the farms that participated in this survey two contributing factors for cross sucking – low milk allowance and bucket feeding [79], [80] –, were highly prevalent, and their combination was found in approximately 40% of the farms. In a study involving 60 of the farms that participated in this survey, Balcão et al. [59] found that high faecal scores (that indicate diarrhoea) were present only in farms with low environmental hygiene scores, with no differences between single and group housing. For many farms in the study region, well managed outdoor, collective housing may be a practical alternative to stimulate early rumen development and grazing behaviour, resulting in high health and welfare standards.

Lastly, a highly relevant finding from this study was the lack of record keeping in most farms. Confirming data from other farms in the same region [81], usable data on calf morbidity and mortality, use of medicines, birth and weaning weights were not available. Our attempt to estimate calves' growth was unsuccessful due to lack of accurate information of calves' birth date. Lack of record keeping is likely to prevent farmers from recognizing calf disease, reduced growth, and mortality as problems in their farms, and may represent an important barrier to changing practices [82]. For example, only a few farmers mentioned diarrhoea and respiratory diseases; farmers also perceived calf mortality rates as “low”, but could not answer precisely how many calves had died in the previous 12 months. Low environmental hygiene scores, combined with the rearing practices reported, highly suggest that farmers underestimate the extent of the problem. Adding to the problem, farmers seemed to fail to perceive the outcome of some practices due to misconceptions (e.g. that 4 L of milk is “adequate” for calves' growth), and low understanding of some issues (e.g. ignoring that pain at dehorning can be minimized by appropriate management, including the use of affordable pharmacological drugs [62]). Greater scientific understanding of the practical, economical and cultural aspects that may play a role in the diffusion (i.e. related to veterinarians and other professional advisors and extensionists) and adoption (i.e. related to farmers) of innovations may help support the needed change.

## Conclusions

We identified a number of management practices that represent risk factors for preweaning calf morbidity and mortality, or may reduce calf welfare: lack of calving and neonate care, early separation from the dam, single housing, low environmental hygiene, insufficient milk allowances, bucket feeding, elective surgical procedures without pain control and culling of a high proportion of newborn males. Farmers justified the choice of practices on practical convenience, short-term economic advantages and traditional conventions, rather than technical knowledge or advice. Altogether, the present data suggest low farmers' interest or awareness of the repercussions of rearing practices for calf wellbeing, survival, growth and future productivity. Thus, to help change the scenario identified in this survey, farmers need to be informed on the production, animal welfare and economic outcomes of neonatal care, calf feeding, housing, and other practices.
